# Whole‐genome methylation analysis of aging human tissues identifies age‐related changes in developmental and neurological pathways

**DOI:** 10.1111/acel.13847

**Published:** 2023-06-12

**Authors:** Ravi Tharakan, Ceereena Ubaida‐Mohien, Christopher Dunn, Mary Kaileh, Rakel Tryggvadottir, Linda Zukley, Chee W. Chia, Ranjan Sen, Luigi Ferrucci

**Affiliations:** ^1^ Translational Gerontology Branch, National Institute on Aging National Institutes of Health Baltimore Maryland USA; ^2^ Laboratory of Genetics and Genomics, National Institute on Aging National Institutes of Health Baltimore Maryland USA; ^3^ Laboratory of Molecular Biology and Immunology, National Institute on Aging National Institutes of Health Baltimore Maryland USA; ^4^ Center for Epigenetics Johns Hopkins University School of Medicine Baltimore Maryland USA

**Keywords:** aging, epigenome, monocytes, skeletal muscle

## Abstract

Age‐associated changes in the DNA methylation state can be used to assess the pace of aging. However, it is not understood what mechanisms drive these changes and whether these changes affect the development of aging phenotypes and the aging process in general. This study was aimed at gaining a more comprehensive understanding of aging‐related methylation changes across the whole genome, and relating these changes to biological functions. It has been shown that skeletal muscle and blood monocytes undergo typical changes with aging. Using whole‐genome bisulfite sequencing, we sought to characterize the genome‐wide changes in methylation of DNA derived from both skeletal muscle and blood monocytes, and link these changes to specific genes and pathways through enrichment analysis. We found that methylation changes occur with aging at the locations enriched for developmental and neuronal pathways regulated in these two peripheral tissues. These results contribute to our understanding of changes in epigenome in human aging.

## Graphical abstract

Human aging is associated with changes in DNA methylation, but the extent of methylation changes across the genome during aging are not understood. Using whole‐genome bisulfite sequencing, we have studied DNA methylation changes in healthy aging in skeletal muscle and monocytes.

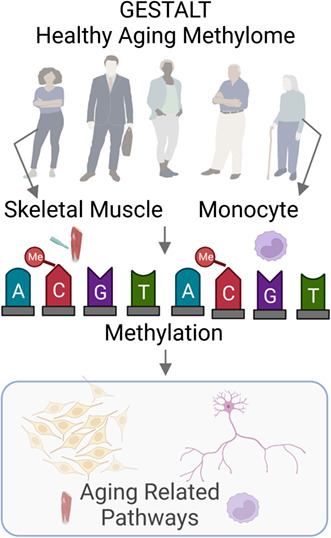

**Abbreviations**
aDMP: aging‐related differentially methylated position
aDMR: aging‐related differentially methylated region
BMI: body‐mass index
DMP: differentially methylated position
DMR: differentially methylated region
GESTALT: Genetic and Epigenetic Signatures of Translational Aging Laboratory Testing
PBMC: peripheral blood mononuclear cells
SWAN: sliding window analysis
TSS: transcription start site
WGBS: whole‐genome bisulfite sequencing

## 1 INTRODUCTION

DNA methylation has become well‐established as a biomarker of human aging and accelerated aging (Horvath & Raj, 2018). Several “epigenetic clocks” have been developed, all of which predict the age of human subjects with high accuracy, as well as independently of chronological age, predicting mortality and other health outcomes (Hannum et al., 2013; Horvath, 2013). Since the original clocks of Horvath and Hannum, some 15 further clocks have been developed (Bergsma & Rogaeva, 2020). The discovery that percent methylation at specific DNA sites changes predictably with aging and conveys information on health status raises the intriguing possibility that DNA methylation may be related to the biological processes that drive human aging (Bell et al., 2019). However, because measures of methylation in human samples are limited to less than 10% of the DNA examined by the current technology, there is still no comprehensive catalog of all DNA methylation changes in human tissues over the course of aging.

Most studies of changes in DNA methylation in human aging use microarrays (Bell et al., 2012; Dobbs et al., 2021; Horvath et al., 2012; Rakyan et al., 2010; Teschendorff et al., 2010), which target a small subset of all CG dinucleotides (Schumacher et al., 2006). While these methods provide information on changes in DNA methylation in many important locations throughout the genome, they are still limited to the set of CpG sites pre‐selected by the manufacturer, and thus will only find changes that occur in these regions. In order to understand how DNA methylation changes generally during aging, we used an unbiased whole‐genome bisulfite sequencing approach, allowing us to quantify DNA methylation across most of the genome. Since DNA methylation is related to cell proliferation and turnover (Beerman et al., 2013), we chose two cell types for profiling with opposite relationships to cell turnover: monocytes turn over frequently, and muscle cells are relatively long‐lived (Gonzalez‐Mejia & Doseff, 2009; Pawlikowski et al., 2015). Furthermore, changes in muscle tissues with aging may be so severe to determine sarcopenia, and changes in monocytes contribute to the decline of immune function with aging (Linton & Thoman, 2014; Moore et al., 2014). Since molecular markers may have several different relationships to age, we performed three different analyses in order to related genome‐wide methylation levels to age. To move toward an understanding the functional correlates of the methylation changes during aging, we then annotated sites and regions whose methylation changes with age as to their nearby genes, under the assumption of local regulation of expression. We then fit those sets of genes into pathways and gene ontology. The results suggest changes in pathways regulating tissue development and tissue specification in both tissues with aging, and surprisingly several pathways that are involved in neuronal function, possibly suggesting the aberrant activation of neuronal pathways in non‐neuronal tissues during aging.

## 2 RESULTS

**FIGURE 1:**
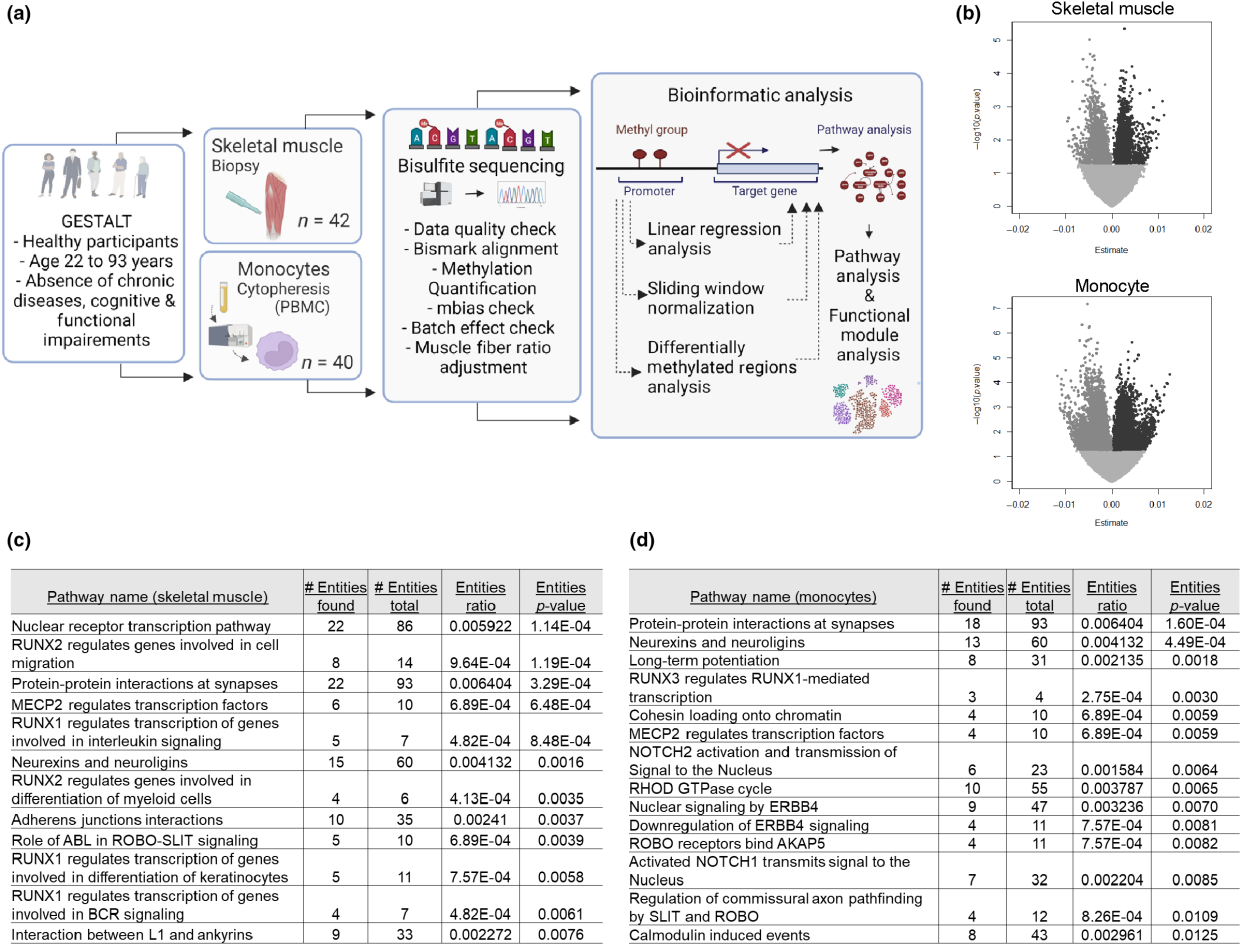
(a) Workflow for this study. In the GESTALT study, health participants were sampled for muscle biopsy and for blood monocytes. DNA was then extracted and whole‐genome bisulfite sequencing was done, with overall quality checks done by FASTQC, alignment and methylation quantification by Bismark, mbias used to detect methylation end biases, adjustment for batch effects and the muscle libraries adjusted for the muscle fiber ratios. Methylation was correlated by three different analyses, and the differentially methylated CpG positions (DMPs) and regions (DMRs) were then linked to genes by their nearest neighbor with 200 base pairs. These genes were then analyzed for pathway enrichment, to determine which pathways are regulated by methylation during aging. (b) Volcano plots for muscle (top) and monocyte (bottom) after linear regression analysis. (c) Pathways associated with aging from linear regression analysis from skeletal muscle. (d) Pathways associated with aging from linear regression analysis from skeletal muscle.

In order to characterize aging‐related DNA methylation changes in skeletal muscle and monocyte samples, we collected muscle biopsies from 43 human subjects and purified monocytes from 40 human subjects. Samples were evenly distributed across ages (Figure S1). We performed whole genome bisulfite sequencing at an approximate read depth of 10× across the genome (Table S1). We then performed three analyses. First, we tested for association with age of all DNA methylation sites using linear regression, to find methylation sites linearly related with age. Next, we used a novel technique called sliding window normalization (Lehallier et al., 2019) which determines methylation changes at each timepoint compared to a neighboring timepoint. Finally, we divided the samples into two groups by age, and determined the changes in DNA methylation comparing young versus old. We use these three analyses to provide a complete picture of methylation changes with aging (Figure 1a).

For both muscle and monocyte samples we performed a linear regression analysis of aging on percent methylation across explored CpGs while adjusting for race, sex, and body mass index (BMI). Because muscle tissue is composed of three fiber types, Type I, Type IIa, and Type IIb, and the ratios between these fiber types change with age; we adjusted the analysis for fiber type ratio using the relative ratio of myosin isoforms in each subject (Ubaida‐Mohien et al., 2019). This analysis resulted in 6780 aging‐associated differentially methylated CpG positions (aDMPs) (*p* < 0.05) in muscle and 29,492 CpGs aDMPs (*p* < 0.05) associated with aging in monocytes (Figure 1b, Tables S2 and S3). Interestingly, the majority of age‐associated CpG sites were intragenic, for both muscle and monocyte, with 62% (4153/6780) intragenic aDMPs for muscle and 57% (16,842/29,492) intragenic aDMPs for monocytes. To gain understanding of the biological pathways that potentially may be impacted by these methylation sites, we first converted aDMPs to genes by finding genes that either overlapped an aDMP (intragenic aDMPs) or genes with a transcription start site (TSS) within 200 bp of the aDMP. We then performed a Reactome pathways analysis on these genes, resulting in a set of pathways which may be regulated by aging‐associated methylation changes (Figure 1). In muscle, the pathways analysis results were dominated by RUNX pathways, as well as MECP2, ROBO, and Notch pathways (Figure 1c). In monocyte, the pathways analysis also showed RUNX, MECP2, ROBO, and Notch, as well as ERBB4 (Figure 1d). Monocyte pathways results also included many neuronal pathways, such as synaptic long‐term potentiation, neuroligins, and axon pathfinding. The muscle pathways also included neuronal pathways, although less prominently. We also compared the results of our WGBS analysis to a previously published array‐based methylation analysis of monocytes (Reynolds et al., 2014), and found that of 1749 age‐related CpG sites found in that paper, only 7 were quantified in our dataset, emphasizing the lack of overlap between shotgun WGBS and microarray data, and the novelty of this dataset.

**FIGURE 2:**
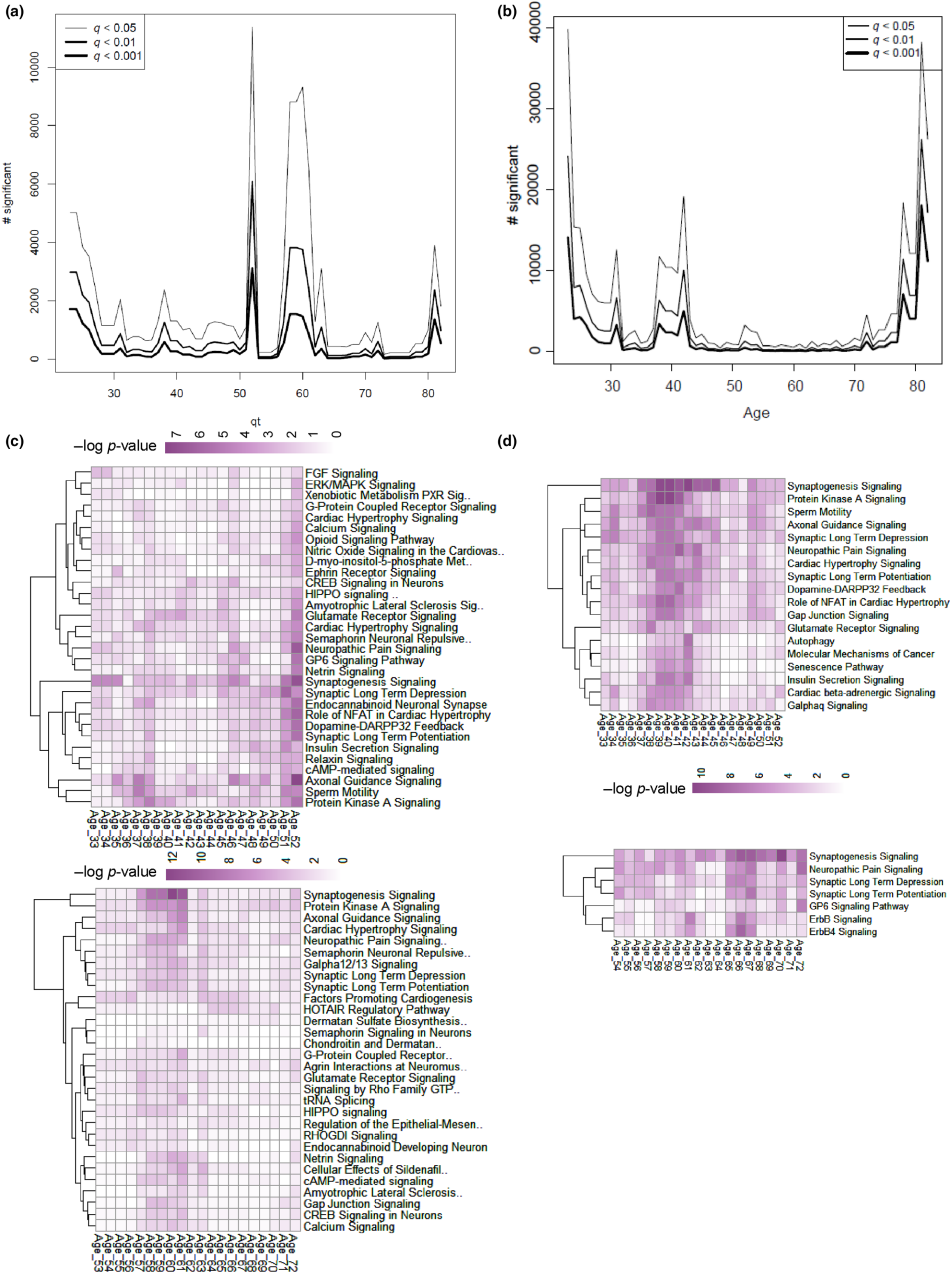
Results of the SWAN analysis. Number of significant CpGs found at each age is plotted for both tissue types, (a) for skeletal muscle and (b) for monocytes. (c, d) Pathways analysis of linked genes for significant CpGs across windows. CpG found to be significant in the SWAN analysis were annotated as to their nearest gene within 200 bp, and these genes were analyzed for pathways enrichment at each timepoint. Analysis was broken into two halves, from ages 33 to 53 (top) and from 53 to 73 (bottom). Each column of the heatmaps shows the pathways that were predominantly regulated by CpG methylation changes at that age.

To understand whether changes in methylation tend to occur homogeneously over the course of the lifetime or are more evident at specific ages, we performed a sliding window analysis (Lehallier et al., 2019). For this method, a 10‐year window is moved along the samples, and the methylation level at each sample is compared to the neighboring 10‐year window. This method determines the methylation changes at each age, showing when in the life course individual CpG sites change in methylation during aging. We first plotted the total number of significantly changed methylation sites between groups at each timepoint (Figure 2a,b). The analysis suggests a peak in aging methylation difference around ages 52 and 62 for muscle (Figure 2a), and earlier peaks of methylation differences for monocytes at approximately ages 33 and 37–42 (Figure 2b). Interestingly, we also reanalyzed our previously published dataset of proteomics data (Ubaida‐Mohien et al., 2019) from muscle samples and we found a similar pattern during the aging time course, with many differences in protein levels becoming more evident in the mid‐50s and 60s (Figure S4). For both monocyte and muscle, we again annotated the CpGs that change with age by taking all CpGs which were changed between any time‐windows across the aging time course, and then for each differential CpG we found any TSS within 200 bp or overlapping a known transcript. This resulted in 18,794 linked transcripts for muscle and 16,490 linked transcripts for monocytes, which were then reduced to their underlying genes. The linked genes were then annotated by a Reactome pathways analysis. The results of the pathways analysis are shown in Figure 2c,d, which show the enriched pathways in the list of significant linked genes for the window centered on each age. It can be seen that the pathways analyses show most changes at the peaks of the graphs of overall changes in Figure 2a,b. We again find that many neuronal pathways appear in these results, in particular in the points in the age timecourse where we see “peaks” in methylation changes. Interestingly, the distances to nearest genes were similarly distributed for the aDMPs for both the linear model and for the SWAN analysis, indicating that aging‐associated DMPs distribute similarly between genic and intergenic regions (Figure S6).

**FIGURE 3:**
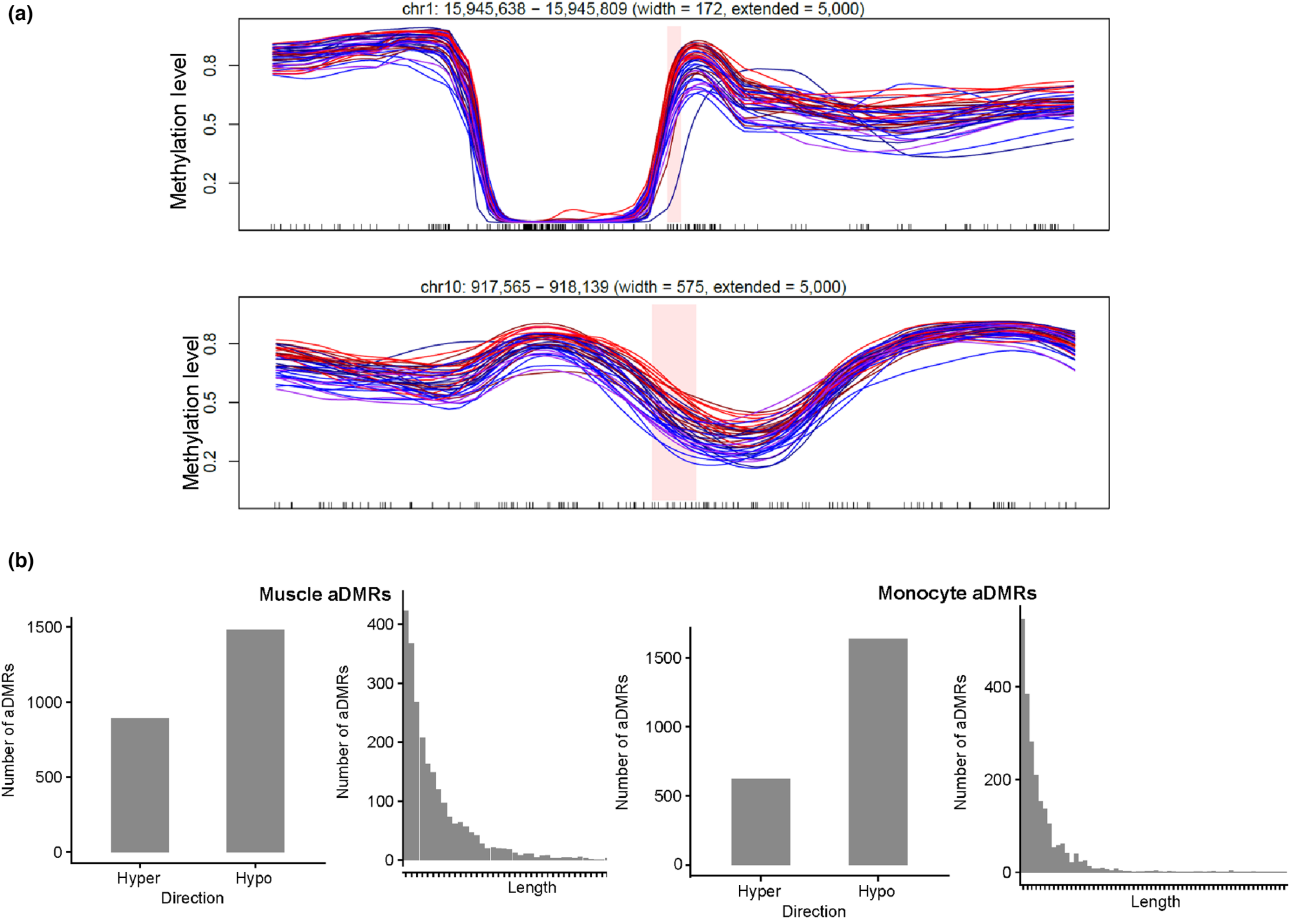
(a) Two examples of aging‐associated differentially methylated regions. (b) Annotation of aDMRs, as to direction of DMR and length of DMR, for both muscle (left) and monocyte (right).

**FIGURE 4:**
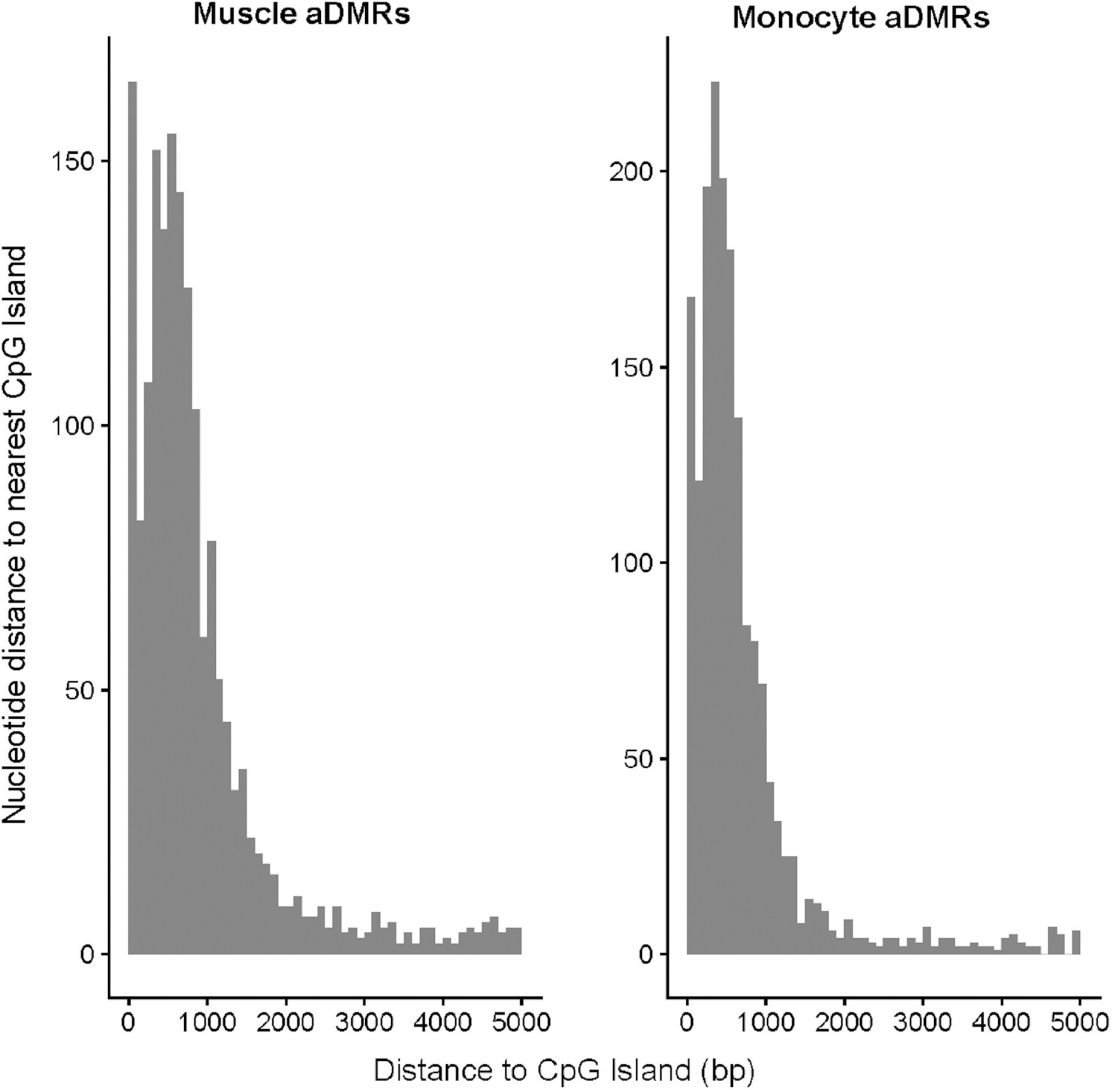
Distribution of distances from aDMRs to nearest CpG islands, showing enrichment of aDMRs in the CpG island shores.

Finally, we utilized the BSmooth algorithm (Hansen et al., 2012), which smooths methylation profiles across the genome, and thus allows the methylation profile for missing CpGs to be imputed from neighboring ones. For this analysis, we divided samples into two groups as evenly sized as possible; for muscle samples, the samples were divided into a younger group of 20 subjects and an older group of 22 subjects, with samples greater than or equal to the age 53 in the older group, and a DMR analysis was performed with a maximum gap between CpGs in a DMR of 300 bp, with neighboring CpGs changed in the same direction grouped into a DMR. The DMR analysis of these muscle samples revealed 2372 aging‐associated differentially methylated regions (aDMRs). For monocytes, the samples were divided into two groups of 20 samples each, with samples greater than age 51 in the older group, and this analysis revealed 2263 aDMRs (Figure 3, Tables S4 and S5). Examples of the DMRs found are shown in Figure 3a, with color‐coding showing clearly the progressive changes in the methylation over the smoothed profiles. The majority of the aDMRs were hypomethylated, and the average length of an aDMR was approximately 6 CpGs (Figure 3b). The aDMRs were found to be enriched in annotated CpG islands or within 2000 bp of a CpG island, in so‐called CpG island shores (Figures 4 and S5). This distribution is similar to that observed by Irizarry et al., in tissue‐specific and cancer‐related DMRs (Irizarry et al., 2009). Interestingly, a majority of the aDMRs were again found to be intragenic, that is, overlapping an annotated transcribed region of the genome, with 73% (1741/2372) and 70% (1589/2263) of the aDMRs intragenic for the muscle and monocyte analyses, respectively. Again annotating these aDMRs as to function, we took all aDMRs either overlapping a transcript or within 200 bp of a transcription start site, giving 1775 aDMRs for muscle and 1634 aDMRs for monocytes. These were annotated as to their nearest transcript (Tables S6 and S7). We then performed pathways and gene ontology analysis on this list of nearest genes to determine the biological processes associated with these CpG sites. Reactome analysis of nearest genes suggested that, in muscle, the Notch pathway is highly represented, while in monocytes, extracellular matrix remodeling and RUNX pathways are highly represented (Tables 1 and 2). In order to annotate these linked genes with more specificity, we also used a pathways analysis comparing against a tissue‐specific gene expression background for both muscle and monocyte (Greene et al., 2015). In muscle, the primary modules found to be overrepresented were muscle contraction, protein translation, and cAMP‐mediated signaling, while in monocytes, 10 modules were found to be overrepresented, including phagocytosis, DNA conformation regulation, and nucleotide metabolism, cell–cell adhesion, and cell migration (Table S8). Surprisingly, the largest overrepresented module in monocyte aDMRs were neuronal development pathways (Table S8). In order to compare the Reactome results to those of another database, we also analyzed this same dataset using KEGG pathways via the Enrichr program (Kanehisa et al., 2017; Kuleshov et al., 2016). This analysis showed very few pathways for each dataset; for muscle, parathyroid hormone synthesis and degradation pathways of amino acids and glycans were prominent; for monocyte, amino acids and glycan pathways were also evident, but also pluripotency, and metabolic pathways such as AMPK and insulin (Table S9).

**TABLE 1:** Top pathways represented in genes which may be regulated by DNA methylation changes over aging in skeletal muscle from DMR analysis. Genes were found which were within 200 bp of aging‐associated DMRs. These genes were then analyzed by Reactome pathways analysis, showing the most highly enriched pathways in the gene set.

Muscle aDMR pathways (Reactome database)				
Pathway name	# Entities found	# Entities total	Entities ratio	Entities *p*‐value
NOTCH3 intracellular domain regulates transcription	9	36	0.0025	0.0053
COPI‐independent Golgi‐to‐ER retrograde traffic	12	63	0.0043	0.0114
NOTCH1 intracellular domain regulates transcription	11	57	0.0039	0.0137
Aggrephagy	9	47	0.0032	0.0254
RHOQ GTPase cycle	11	63	0.0043	0.0261
NOTCH4 intracellular domain regulates transcription	6	26	0.0018	0.0291
RHOB GTPase cycle	12	75	0.0052	0.0367
RHO GTPases activate IQGAPs	7	36	0.0025	0.0424
PTK6 promotes HIF1A stabilization	3	9	6.20E‐04	0.0461

**TABLE 2:** Top pathways represented in genes which may be regulated by DNA methylation changes over aging in monocytes from DMR analysis. Genes were found which were within 200 bp of aging‐associated DMRs. These genes were then analyzed by Reactome pathways analysis, as in Table 1.

Muscle aDMR pathways (Reactome database)				
Pathway name	# Entities found	# Entities total	Entities ratio	Entities *p*‐value
Elastic fiber formation	11	46	0.003168	0.0015
Regulation of cortical dendrite branching	3	4	2.75E‐04	0.0043
Vasopressin‐like receptors	3	6	4.13E‐04	0.0131
TNFR1‐mediated ceramide production	3	6	4.13E‐04	0.0131
RUNX2 regulates genes involved in differentiation of myeloid cells	3	6	4.13E‐04	0.0131
Molecules associated with elastic fibers	8	38	0.002617	0.0131
A tetrasaccharide linker sequence is required for GAG synthesis	7	31	0.002135	0.0139
Regulation of signaling by NODAL	4	12	8.26E‐04	0.0169
SUMOylation of intracellular receptors	8	40	0.002754	0.0172
Regulation of gene expression by hypoxia‐inducible factor	4	15	0.001033	0.0343
ChREBP activates metabolic gene expression	3	9	6.20E‐04	0.03713
FRS‐mediated FGFR1 signaling	6	31	0.002135	0.0419
RUNX3 regulates p14‐ARF	4	16	0.001102	0.0418
RUNX3 regulates RUNX1‐mediated transcription	2	4	2.75E‐04	0.0419
Collagen formation	14	104	0.007162	0.0459
Collagen biosynthesis and modifying enzymes	11	76	0.005233	0.0472
SOS‐mediated signalling	3	10	6.89E‐04	0.0480

**FIGURE 5:**
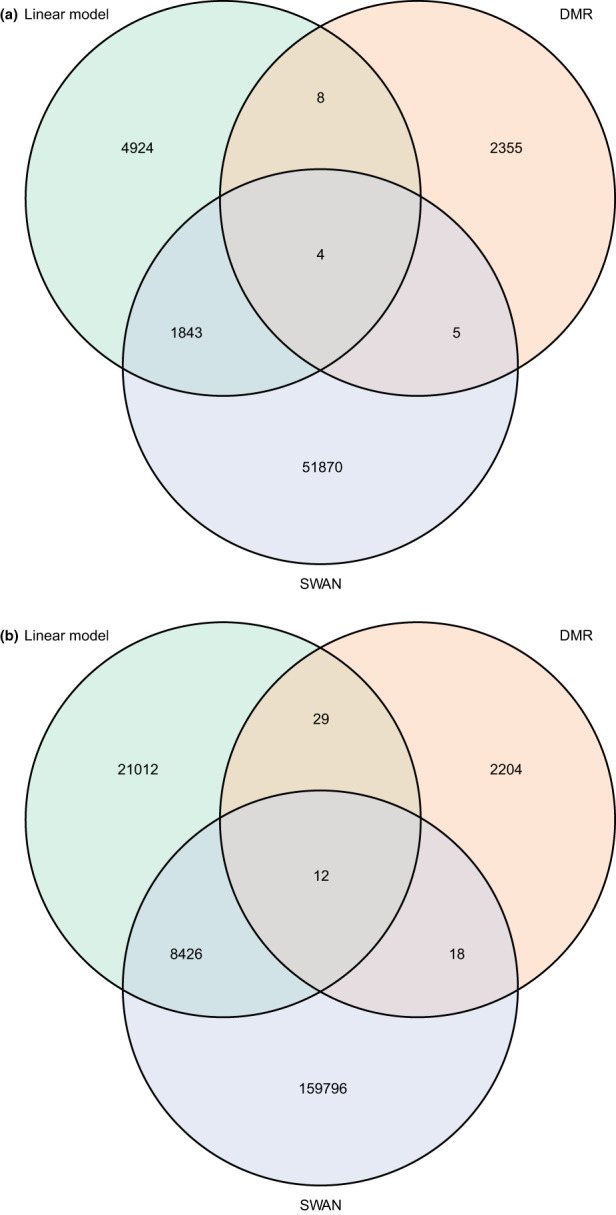
Overlap between the three analysis methods, showing CpG associated with age in common between all three, for (a) muscle and (b) monocyte.

In order to determine the relationship between the three types of analyses we performed, we compared the sets of CpGs found by the three analyses. The results of this comparison are shown in Figure 5. For muscle, 4 CpGs were found to be in common between all three analyses, and for monocyte, 12 CpGs were found to be in common. Annotations of these common CpGs are found in Table S10. Interestingly, most of the common CpGs were in CpG island shores or shelves, either within the gene in an exon or intron, or upstream in the promoter region of a gene.

## 3 DISCUSSION

A strength of our study is the use of whole‐genome bisulfite sequencing, which allows an “unbiased” look at the methylation state of the entire genome, as compared to microarray experiments. We believe that our study shows the power of this unbiased method to discover novel biology in the aging methylome, despite this technique being more expensive. However, array‐based methods, since they use targeted probes, may provide more accurate and consistent quantitative information, as compared to WGBS, which can only quantify methylation by counting the number of times that a methylated CpG was sequenced, using this count as a proxy of the total population of cells in a sample. A weakness of our study is the relatively low depth at which our samples were sequenced, due to resource constraints, which allowed quantification of a smaller subset of the methylome than might otherwise be possible.

In recent years, many “epigenetic clocks” have been constructed, all of which are able to predict age given a set of CpGs and their methylation state (Bergsma & Rogaeva, 2020). Due to the striking correlation between age and methylation state, several limited clocks, with low predictive power, were described (Weidner et al., 2014); but the major breakthrough in the field was the machine learning‐based method of Horvath (Horvath, 2013), which leveraged large amounts of data to train a classifier which was robust to tissue type and highly predictive of age. Similarly, the Hannum clock (Hannum et al., 2013) also uses a similar regression method to derive a clock. However, there is little overlap between these two clocks, suggesting that much of the genome may be related to, and predictive of, age, beyond that captured by known epigenetic clocks. Beyond general clocks, there are also specific “biological clocks”, which forgo the unbiased approach of previous clocks and rather target the methylation states of genes and biological systems known to be involved in aging, such as the Polycomb genes or the nucleolus (Wang & Lemos, 2019; Yang et al., 2016; Zhang et al., 2017). These suggest that specific biological pathways are involved in the regulation of epigenetic clocks.

We have recently published a study of trascriptomic changes during aging, where we found that the gene TET2, a central regulator of DNA methylation states, increases with age (Tumasian 3rd et al., 2021), which could suggest that DNA methylation is generally changing in aging cells. In this study, we have studied the DNA methylome of two tissues which undergo major changes during aging. Skeletal muscle has been shown to lose mass during aging, and the resulting loss of muscle strength causes many pathologies of aging (Moore et al., 2014). Similarly, the immune system is believed to lose function during aging, in so‐called immunosenescence, and we therefore studied one component of the innate immune system, the blood monocyte compartment. DNA methylation has also been shown to be a good biomarker for age throughout the human body, although its mechanistic relationship to aging is not understood (Horvath & Raj, 2018).

DNA methylation can change in two ways: either methylation can change during cell division, due to errors when methylation states are copied from parent to daughter cell (Kim et al., 2005); or transcription factors can remodel the DNA, removing or adding methyl groups (Zhu et al., 2016). Interestingly, we find a substantial enrichment of aDMRs in the shores of CpG islands. These island shores have previously been described to be associated with cell and tissue specification, as well as progression to cancer (Doi et al., 2009; Irizarry et al., 2009). Thus, our set of aDMRs suggests that regulation of cell tissue specification may become disrupted in aging, which may predispose aged individuals to cancer (Goodell & Rando, 2015). CpG islands are associated with approximately 70% of all human transcription start sites, including most constitutively active genes as well as those involved in tissue specification (Deaton & Bird, 2011; Grand et al., 2021). Thus, methylation changes in CpG island shores may represent a subset of aging‐associated methylation changes which are directly caused by transcription factor binding. These transcription factors could be expected to be involved in tissue development and specification, such as Notch (Siebel & Lendahl, 2017) and RUNX (Mevel et al., 2019), as we find in our pathways analysis. Importantly, the developmental pathways Notch, RUNX, Robo, and MECP2 appear in both our monocyte and muscle analyses, suggesting common pathways involved in methylation changes.

Several of our analyses find evidence of differential methylation of neuronal pathways during aging in tissues which are not expected to have substantial neuronal cell populations, monocytes, and skeletal muscle. The reasons for these changes are unknown, but the modulation of neuronal cell signaling pathways in peripheral tissues could be a dysfunctional effect of the aging of the central nervous system. For example, heterochronic parabiosis experiments have shown that soluble factors can rejuvenate tissues throughout the body, suggesting that secreted factors play an important role in aging; similarly, the senescence‐associated secretory phenotype is a set of secreted factors, produced by senescent cells during aging, which may “spread” aging through the body (Birch & Gil, 2020; Katsimpardi et al., 2014). There may thus be some communication between nervous system and periphery which may be involved in neuronal pathway activation.

## 4 METHODS

### 4.1 Human subject selection and sample collection

Briefly, muscle biopsies and monocyte samples were collected from participants in the genetic and epigenetic study of aging and laboratory testing (GESTALT) study. Inclusion criteria for muscle are as in Ubaida‐Mohien et al. (2019) and in Schrack et al. (2014) and for monocyte are as in Roy et al. (2022). All participants provided informed written consent at each new visit, and the study was approved by the Institutional Review Board of NIEHS and conducted within the Clinical Research Core of the NIA. For muscle, 42 samples were available for this study; for monocyte, 40 samples were available. Muscle biopsies were prepared as in Ubaida‐Mohien et al. (2019). Briefly, the depth of subcutaneous fat was determined by MRI, an area of the vastus lateralis muscle was anaesthetized and muscle biopsies were obtained using a 6‐mm Bergstrom needle by standard methods. For monocytes samples, monocytes were negatively enriched from the donors PBMCs using “EasySep Human Monocyte Enrichment Kit w/o CD16” (StemCell Technologies) following the manufacturer's instructions. After enrichment, monocytes were stained with anti CD14 antibody (Biolegend) and then sorted on a BD FACSAria II (Becton Dickinson). The FACS‐sorted monocytes were washed with PBS, snap frozen, and then stored in −80°C for subsequent DNA isolation. All enriched and sorted cells were >95% pure determined by flow cytometry.

### 4.2 DNA extraction, library preparation, and sequencing

Monocytes DNA was isolated from 1–2 million cells using Qiagen DNeasy kit (Genetic Resources Core Facility, JHU, Baltimore). DNA was extracted from skeletal muscle biopsies using DNAQuik DNA extraction protocol (Reprocell). Whole genome bisulfite sequencing single indexed libraries were generated using NEBNext Ultra DNA library Prep kit for Illumina (New England BioLabs) according to the manufacturer's instructions with modifications. 500 ng input gDNA was quantified by Qubit dsDNA BR assay (Invitrogen) and spiked with 1% unmethylated lambda DNA (Promega, cat # D1521) to monitor bisulfite conversion efficiency. Input gDNA was fragmented by Covaris S220 Focused‐ultrasonicator to an average insert size of 350 bp. Samples were sheared for 60 s using Covaris microTUBES, instrument was set to duty cycle 10%, intensity 5, and cycles per burst 200. Size selection was performed using AMPure XP beads and insert sizes of 300–400 bp were isolated. Samples were bisulfite converted after size selection using EZ DNA Methylation‐Lightning Kit (Zymo cat#D5030) following the manufacturer's instructions. Amplification was performed after the bisulfite conversion using Kapa Hifi Uracil+ (Kapa Biosystems, cat# KK282) polymerase using the following cycling conditions: 98°C 45 s/8 cycles: 98°C 15 s, 65°C 30 s, 72°C 30 s/72°C 1 min. AMPure cleaned‐up libraries were run on 2100 Bioanalyzer (Agilent) High‐Sensitivity DNA assay, samples were also run on Bioanalyzer after shearing and size selection for quality control purposes. Libraries were quantified by qPCR using the Library Quantification Kit for Illumina sequencing platforms (KAPA Biosystems, cat#KK4824) using 7900HT Real Time PCR System (Applied Biosystems). WGBS libraries were sequenced on an Illumina HiSeq4000 instrument using 150 bp paired‐end indexed reads and 10% of non‐indexed PhiX library control (Illumina).

### 4.3 Sequencing quality control and alignment

Deep sequencing data quality was checked with FastQC, with all sequencing runs passing with expected quality (Supplemental Table S1). The Illumina adaptor sequence AGATCGGAAGAGC was trimmed, and reads with Phred score <20 were filtered, using TrimGalore 0.4.2 and Cutadapt 1.14. The maximum trimming error rate was set to 0.1 and the minimum final length was set at 20 bp. Paired‐end alignment was performed on hg19 (GRCh37) with Bismark 0.17.0, samtools 1.5, and bowtie 2.2.9. Genome was converted and indexed using Bisulfite Genome Indexer 0.16.0. M‐bias analysis was performed according to Hansen et al. (2012), and showed evidence of end‐repair bias (Figure S1a), and therefore 2 bases were dropped from the 5′ end of read 2 (–ignore_r2 2); m‐bias plots also showed evidence of overlapping reads (Figure S1b), so 5 bases were dropped from the 3′ end of both read 1 and read 2 (–ignore_3prime 5 –ignore_3prime_r2 5), for generation of genome‐wide cytosine reports. Batch effects were checked and batch‐affected methylation sites were imputed with BEclear (Akulenko et al., 2016), with very few CpGs (<1% in all samples) affected by batch. Muscle fiber ratio was calculated as the ratio Myosin 7/(Myosin 1 + Myosin 2 + Myosin 4), and used to adjust models.

### 4.4 Differentially methylated regions analysis

Cytosine reports were read into the bsseq 1.22.0 package in R (Hansen et al., 2012). Reads were assigned to chromosomes and smoothing was performed separately for each chromosome. Smoothing was performed with the following parameters: minimum number of methylation loci in smoothing window of 70; minimum smoothing window of 1000 bases; and maximum gap between two loci for smoothing of 10^8^. To determine differentially methylated regions, a *t*‐statistic was calculated at each methylation locus by the difference in means between the young group and the old group divided by the standard deviation, using an estimate of the baseline variance. As variability is believed to increase with age (Jenkinson et al., 2017), baseline variance was taken from the young group, and as the marginal distribution of the t‐statistic showed a small amount of large‐scale methylation change (Figure S2), local mean‐correction was used. We selected a cutoff of ±4.6 for the extreme values of the t‐statistic. Methylation sites farther than 300 base pairs apart were broken into separate DMRs. Only CpGs covered at least 2× in all samples were included in the analysis. For annotation, hg19 transcript locations were obtained through Bioconductor 3.10 through TxDb.Hsapiens.UCSC.hg19.knownGene and transcription start sites were determined. CpG island locations were obtained from AnnotationHub (identifier AH5086, snapshot 2019‐10‐29). Degree of overlap with and distance to transcription start sites and CpG island were determined using GRanges. Pathways analysis was performed through Reactome, by obtaining all genes >200 bp from a TSS, then loading the list into the Reactome database. A functional module analysis was performed through HumanBase in the same manner (Zhou & Troyanskaya, 2015).

### 4.5 Linear regression analysis

For linear regression, each CpG was considered individually and only CpG covered at least 10× in all samples were included. Age beta coefficients were calculated using a mixed linear regression model on the percent methylation for each CpG site adjusted on sex, race, and BMI. LmerTest was used for calculation of *p*‐values and all methylation sites *p* < 0.05 were taken to be age‐associated. For pathways analysis, CpG sites were annotated as to the nearest transcription start site within 200 bp as above, and the resulting set of genes was analyzed using Reactome pathways analysis. For muscle, all sites *p* < 0.05 were included in the pathways analysis, while for monocytes, due to the high number of significant CpGs, sites *p* < 0.01 were included.

### 4.6 Sliding window normalization

For sliding window normalization, single CpG analysis was performed as above and CpGs were filtered at 10× coverage for all samples. Window was set at a width of 10 years and progressed by 1 year at a time. Differential expression was tested on a linear model with two variables (Lehallier et al., 2019). Overall profiles were plotted as the total number of CpGs significantly differentially methylated at each window, with Benjamini–Hochberg correction (*q* < 0.05). For pathways analysis, CpGs were again annotated as above, and the nearest gene within 200 bp found, and these genes loaded into Ingenuity Pathways analysis. Due to computational constraints, 20 timepoints could be analyzed at a time, and thus the timepoints were divided into groups from ages 33 to 53 and again from 53 to 72. Since the oldest and youngest timepoints would include ages where there is no data, and thus report many artifactual significant hits, these timepoints (<33 and >72) were excluded. Pathways were filtered with Benjamini–Hochberg correction for significance. For the muscle analysis, the significance threshold was set to *p* < 0.01, and for monocyte, the significance threshold was set to *p* < 0.0001.

## AUTHOR CONTRIBUTIONS

Luigi Ferrucci and Ranjan Sen conceived the study and designed experiments; Luigi Ferrucci, Ravi Tharakan, and Ceereena Ubaida‐Mohien performed analysis; Ravi Tharakan, Ranjan Sen, Ceereena Ubaida‐Mohien, and Luigi Ferrucci wrote the paper; Mary Kaileh, Christopher Dunn, Rakel Tryggvadottir, Linda Zukley, and Chee W. Chia performed experiments and assisted in the writing of the paper.

## CONFLICT OF INTEREST STATEMENT

None declared.

## Supporting information


**Figure S1.** Overall distribution of samples according to age. Subjects ranged from ages 22 to 83.
**Figure S2.** Example m‐bias plots for muscle samples from one subject (NIH‐304). M‐bias plot was generated from Bismark methylation extractor with no bases dropped. Read 2 (a) shows substantial variability at the 5′ end, suggesting end‐repair bias; therefore 3′ bases were dropped from the 5′ end of read 2. Read 1 (b) shows little variability, but 5 bases were therefore dropped from the 3′ end of both read 1 and read 2 to guard against read overlap.
**Figure S3.** Example *t*‐statistic distribution from muscle samples for chromosome 1. A *t*‐statistic was calculated for all CpGs in the skeletal muscle sample and the subset mapping to chromosome 1 were plotted. Shown is both the distribution of the *t*‐statistic both before and after local mean correction.
**Figure S4.** Results of SWAN analysis of proteomics data from muscle samples. Number of significant CpGs found at each age is plotted for skeletal muscle.
**Figure S5.** Distribution of distances from aDMRs to nearest CpG islands, showing enrichment of aDMRs in the CpG island shores. Also shown is all CpGs in the genome as background, showing enrichment of aDMRs in shore regions over background.
**Figure S6.** Distribution of distances from aDMPs to nearest gene for the aDMPs derived from the linear model and from the SWAN analysis.

**Table S1.** Read depth and coverage for all WGBS samples.

**Table S2.** Linear regression results from all muscle samples.

**Table S3.** Linear regression results from all monocyte samples.

**Table S4.** Muscle differentially methylated regions.

**Table S5.** Monocyte differentially methylated regions.

**Table S6.**Annotations for muscle aDMRs.

**Table S7.** Annotations for monocyte aDMRS.

**Table S8.** HumanBase analysis results.

**Table S9.** Muscle and monocyte KEGG analysis results.

**Table S10.** Common aDMPs for all analysis methods for muscle and monocyte.

Table S11.

## Data Availability

The data (bisulfite sequencing datasets) that support the findings of this study are available on request from the authors.
