# Supplementary material for: Whole‐genome methylation analysis of aging human tissues identifies age‐related changes in developmental and neurological pathways
Source: Aging Cell. 2023 Jun 12;22(7):e13847. doi: 10.1111/acel.13847 (PMC10352543; doi:10.1111/acel.13847)
Supplement: Supplementary file 1 — Figure S1. Overall distribution of samples according to age. Subjects ranged from ages 22 to 83. Figure S2. Example m‐bias plots for muscle samples from one subject (NIH‐304). M‐bias plot was generated from Bismark methylation extractor with no bases dropped. Read 2 (a) shows substantial variability at the 5′ end, suggesting end‐repair bias; therefore 3′ bases were dropped from the 5′ end of read 2. Read 1 (b) shows little variability, but 5 bases were therefore dropped from the 3′ end of both read 1 and read 2 to guard against read overlap. Figure S3. Example t‐statistic distribution from muscle samples for chromosome 1. A t‐statistic was calculated for all CpGs in the skeletal muscle sample and the subset mapping to chromosome 1 were plotted. Shown is both the distribution of the t‐statistic both before and after local mean correction. Figure S4. Results of SWAN analysis of proteomics data from muscle samples. Number of significant CpGs found at each age is plotted for skeletal muscle. Figure S5. Distribution of distances from aDMRs to nearest CpG islands, showing enrichment of aDMRs in the CpG island shores. Also shown is all CpGs in the genome as background, showing enrichment of aDMRs in shore regions over background. Figure S6. Distribution of distances from aDMPs to nearest gene for the aDMPs derived from the linear model and from the SWAN analysis. [file ACEL-22-e13847-s012.pdf]

Supplemental Figures

Whole-genome Methylation Analysis of Aging Human Tissues Finds Changes in Developmental and Neurological Pathways

Supplemental Figure 1

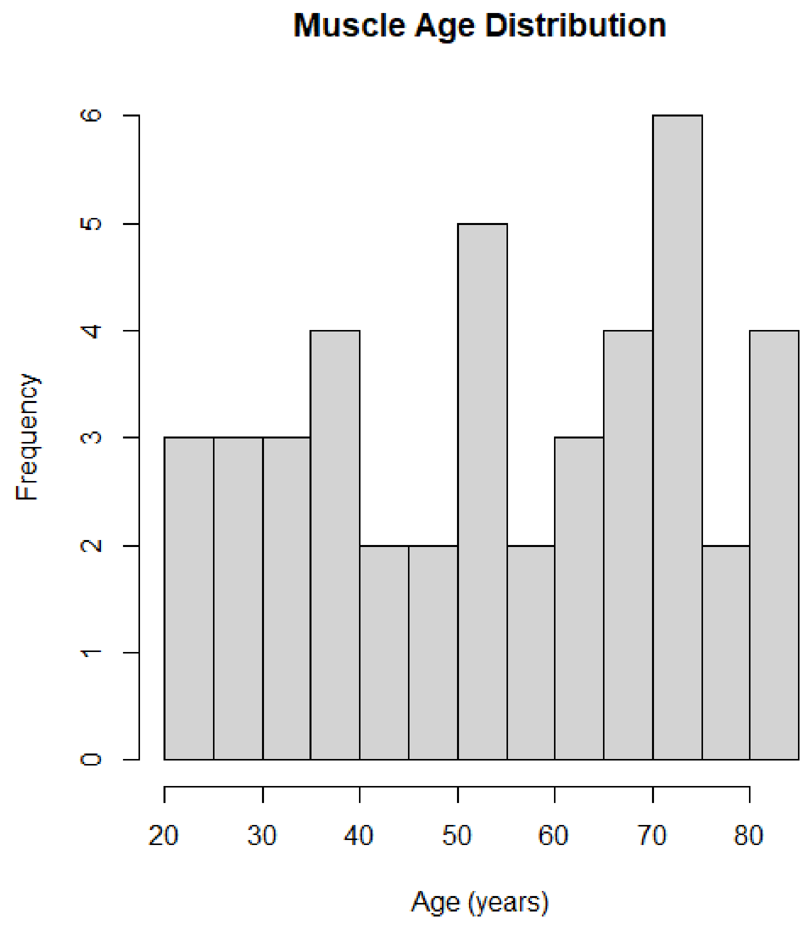

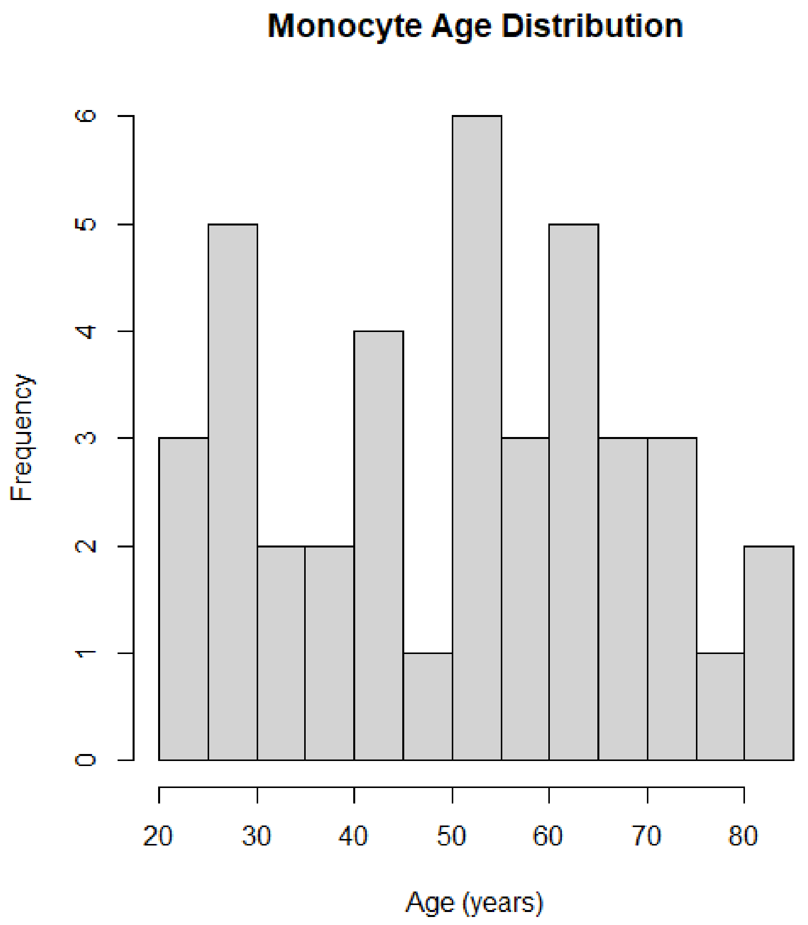

Supplemental Figure 2

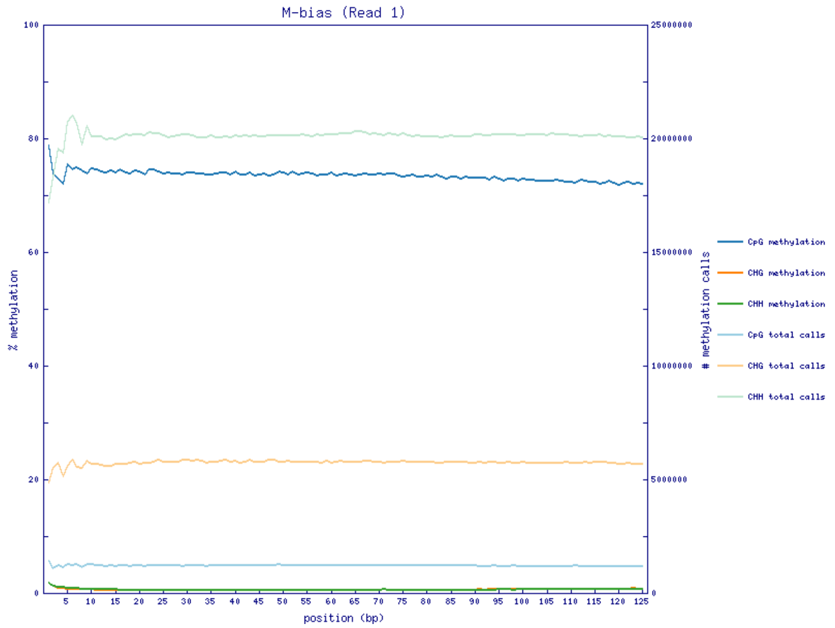

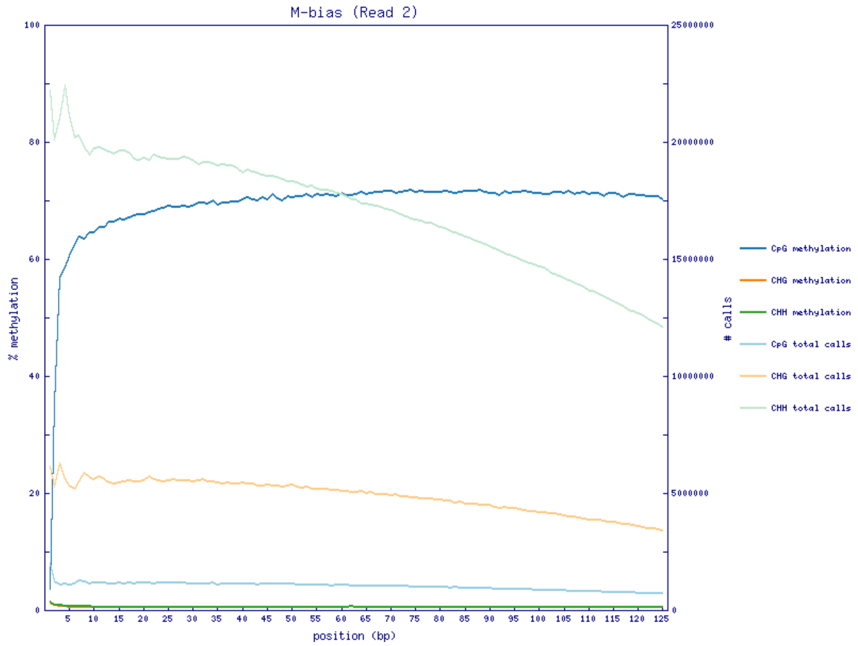

Supplemental Figure 3

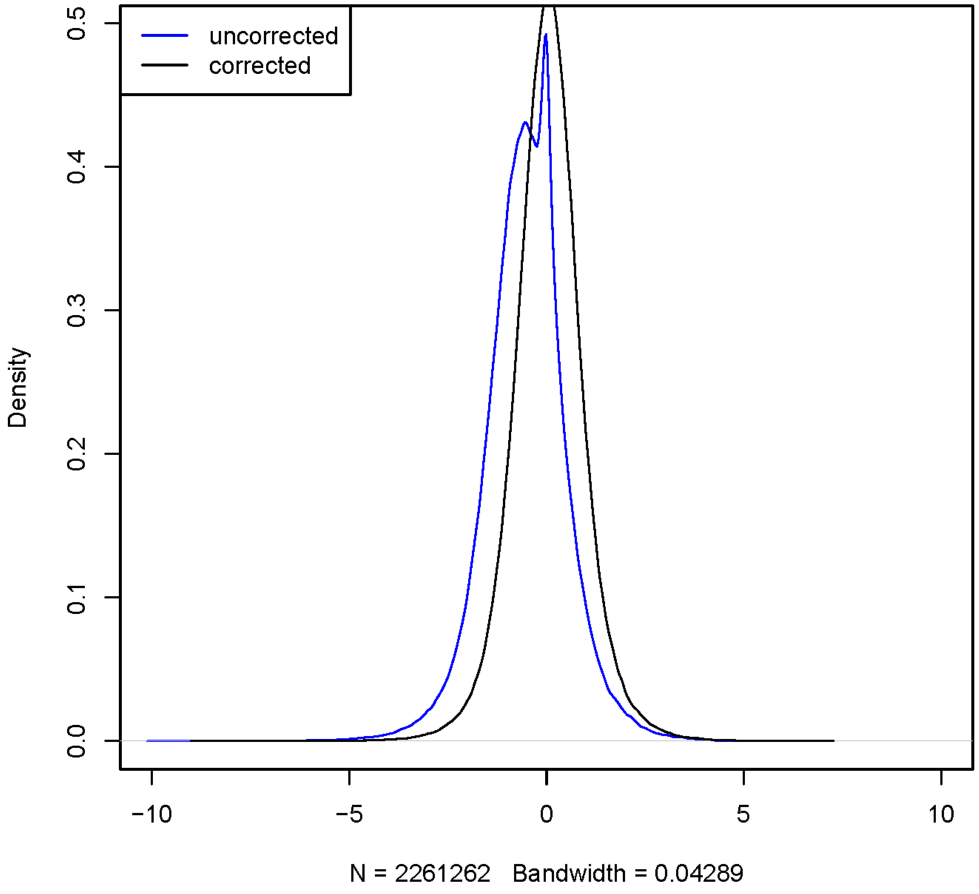

Supplemental Figure 4

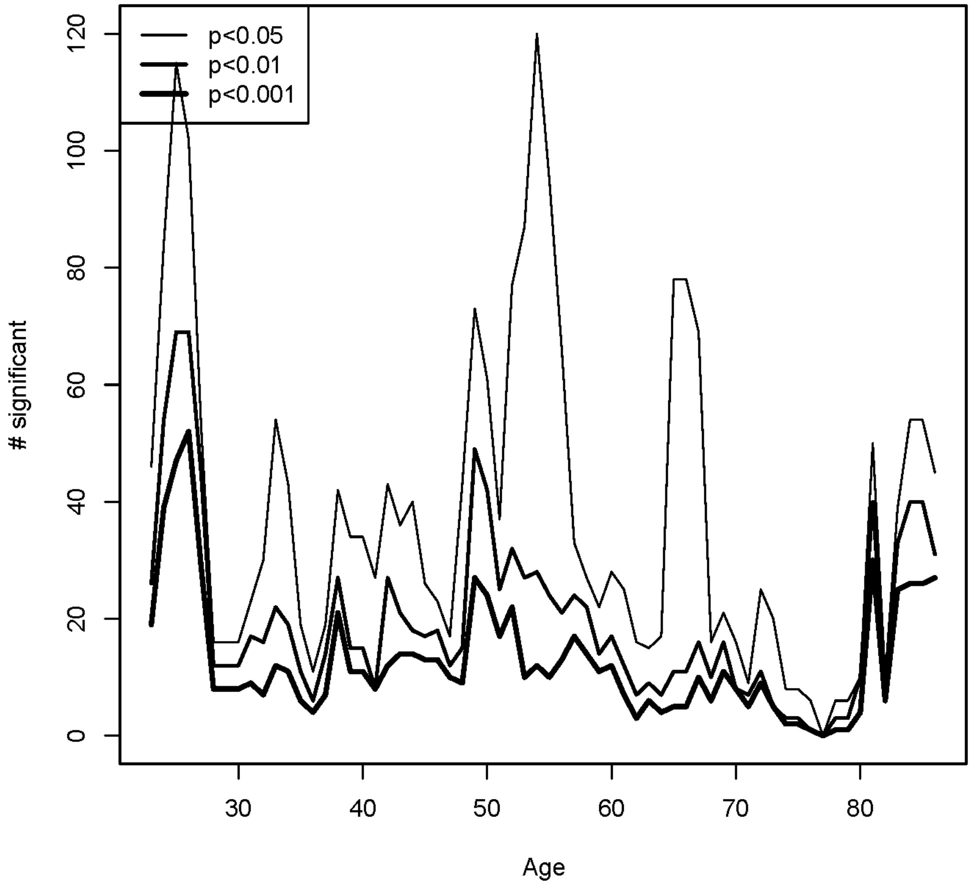

Supplemental Figure 5

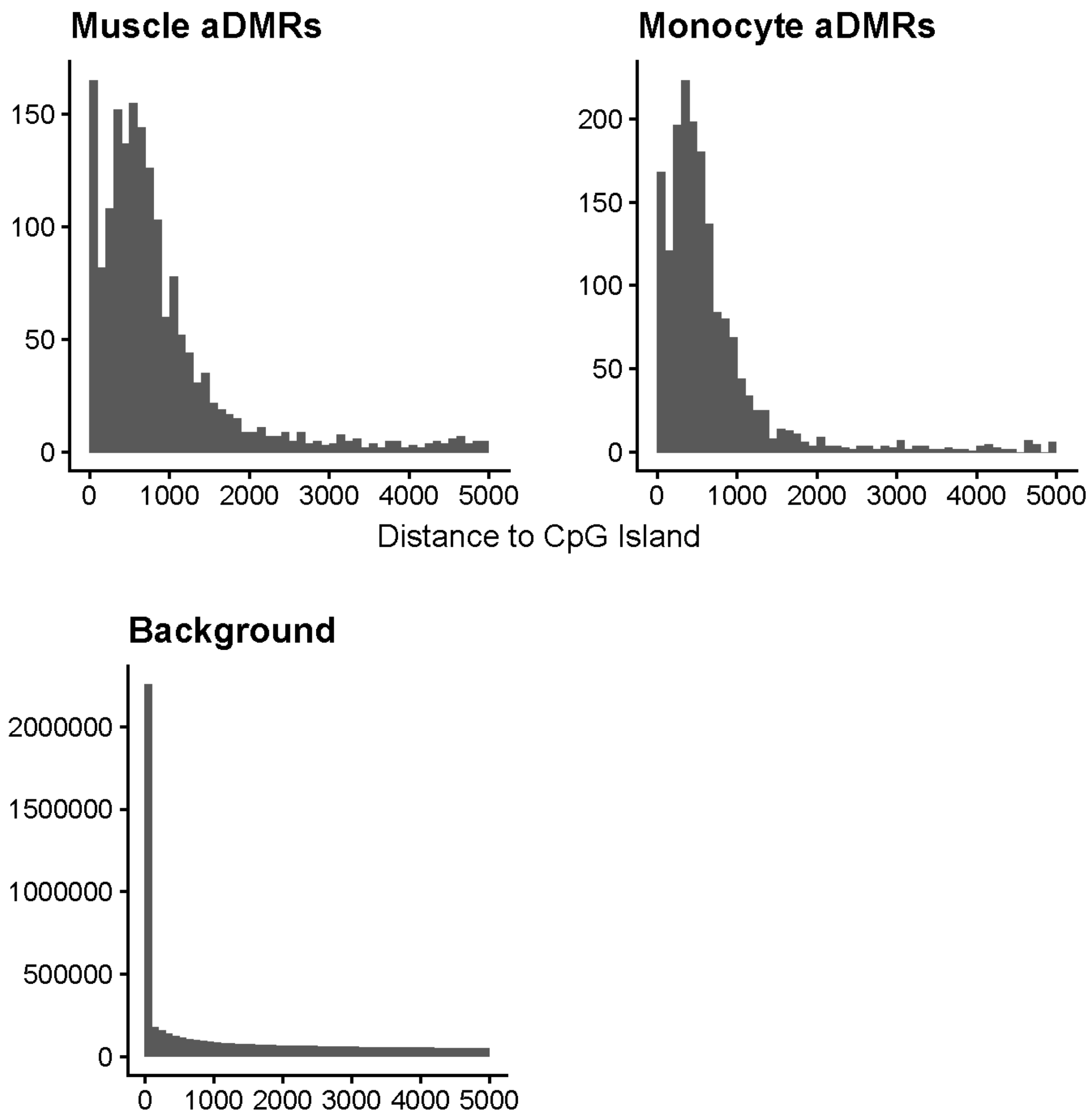

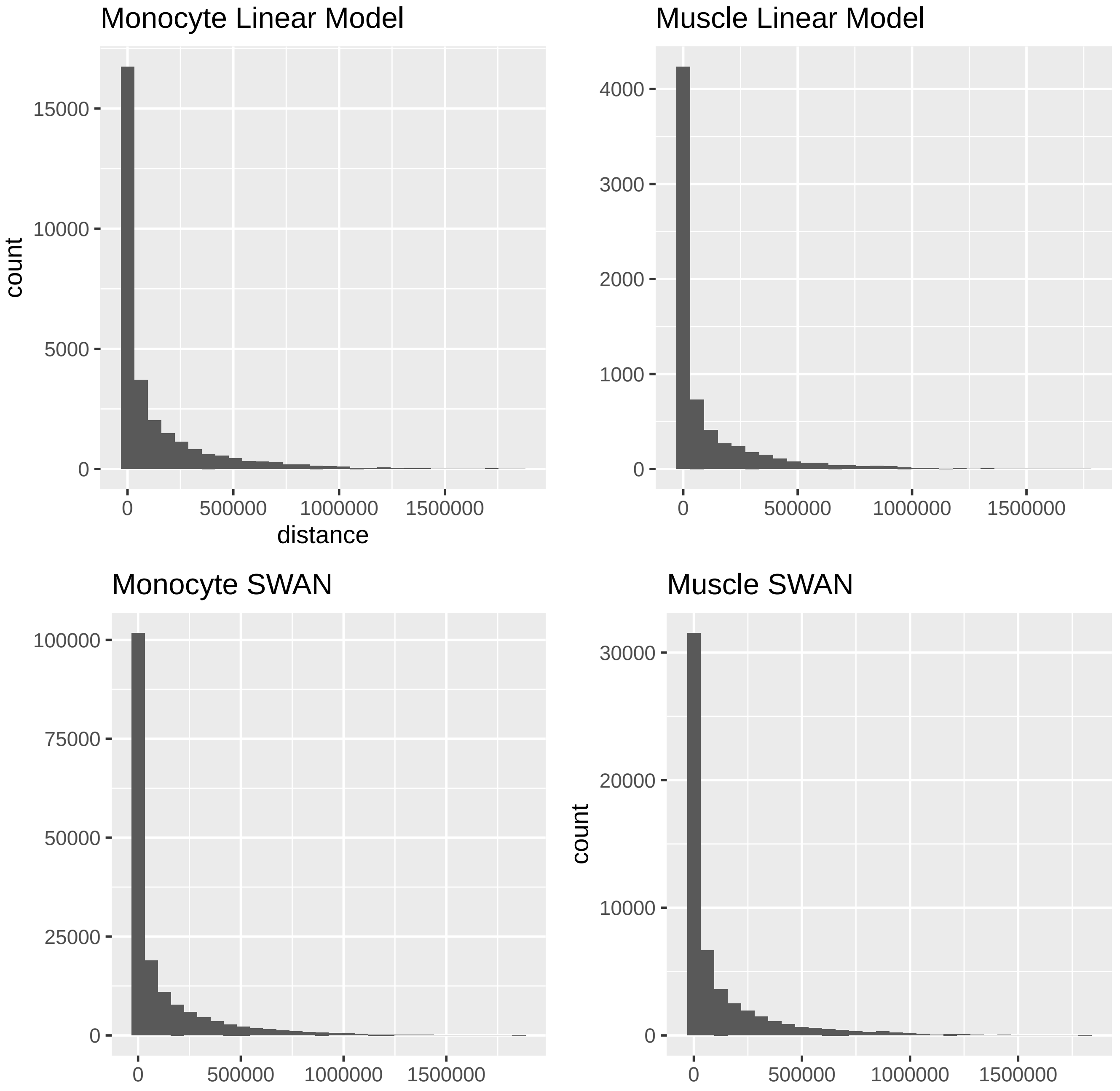
Monocyte Linear Model
Muscle Linear Model
Monocyte SWAN
Muscle SWAN
count
distance
0
5000
10000
15000
1000
2000
3000
4000
25000
50000
75000
100000
20000
30000
500000
1000000
1500000
