# Supplementary material for: Whole‐genome methylation analysis of aging human tissues identifies age‐related changes in developmental and neurological pathways
Source: Aging Cell. 2023 Jun 12;22(7):e13847. doi: 10.1111/acel.13847 (PMC10352543; doi:10.1111/acel.13847)
Supplement: Supplementary file 9 — Table S8. HumanBase analysis results. [file ACEL-22-e13847-s003.pdf]

Muscle

MODULE	TOP TERMS (Max 10)	Q VAL	GENES	TERMS
M1	muscle contraction	0.00324803	231	91
	muscle system process	0.00470446		
	striated muscle contraction	0.00808275		
	cardiac muscle cell action potential	0.01347722		
	monovalent inorganic cation transport	0.01548003		
	regulation of heart rate	0.02577981		
	regulation of system process	0.02631673		
	action potential	0.03109605		
	membrane depolarization during cardiac muscle cell action potential	0.03297837		
	sodium ion transmembrane transport	0.03321690		
M2	peptide biosynthetic process	0.00324803	153	87
	regulation of translation	0.00324803		
	viral translational termination-reinitiation	0.00324803		
	peptide metabolic process	0.00370752		
	regulation of cellular amide metabolic process	0.00470446		
	amide biosynthetic process	0.00487713		
	translation	0.00547151		
	IRES-dependent viral translational initiation	0.00808275		
	posttranscriptional regulation of gene expression	0.01347722		
	viral translation	0.01379066		
M3	membrane protein proteolysis	0.00324803	2	2
	membrane protein ectodomain proteolysis	0.00324803		
M4	signal transduction in response to DNA damage	0.00370752	2	2
	response to radiation	0.01207883		
M5	endomembrane system organization	0.01207883	2	2
	positive regulation of cellular component biogenesis	0.01571702		
M6	cAMP-mediated signaling	0.04658001	119	12
	oligosaccharide metabolic process	0.04658001		
	regulation of cAMP-mediated signaling	0.04658001		
	cyclic-nucleotide-mediated signaling	0.04818862		
	positive regulation of protein tyrosine kinase activity	0.07160169		
	positive regulation of peptidyl-tyrosine phosphorylation	0.07901231		
	peptidyl-tyrosine phosphorylation	0.09482958		
	response to organophosphorus	0.09482958		
	peptidyl-tyrosine modification	0.09627148		
	response to purine-containing compound	0.09627148		
M7	negative regulation of cell cycle	0.04876926	6	1

Monocytes

MODULE	TOP TERMS (Max 10)	Q VAL	GENES	TERMS
M1	phagocytosis	0.00076006	22	136
	calcitriol biosynthetic process from calciol	0.00252275		
	cellular alcohol biosynthetic process	0.00252275		
	cellular alcohol metabolic process	0.00252275		
	regulation of calcidiol 1-monooxygenase activity	0.00252275		
	vitamin D3 metabolic process	0.00252275		
	extrinsic apoptotic signaling pathway via death domain receptors	0.00252275		
	cellular response to nicotine	0.00252275		
	fat-soluble vitamin biosynthetic process	0.00274453		
	regulation of vitamin D biosynthetic process	0.00274453		
M2	cardiac muscle cell-cardiac muscle cell adhesion	0.00274453	45	43
	bundle of His cell-Purkinje myocyte adhesion involved in cell communication	0.00274453		
	regulation of ventricular cardiac muscle cell action potential	0.00407265		
	bundle of His cell to Purkinje myocyte communication	0.00511131		
	regulation of cardiac muscle cell action potential	0.00624671		
	regulation of cardiac muscle cell contraction	0.00690591		
	regulation of actin filament-based movement	0.00721783		
	ventricular cardiac muscle cell action potential	0.00823837		
	regulation of action potential	0.00899977		
	homotypic cell-cell adhesion	0.01030412		
M3	DNA conformation change	0.00274829	17	12
	DNA duplex unwinding	0.00487895		
	DNA geometric change	0.00540932		
	mitotic sister chromatid segregation	0.01443071		
	sister chromatid segregation	0.01651909		
	ncRNA processing	0.01855751		
	nuclear chromosome segregation	0.01931505		
	mitotic nuclear division	0.02327512		
	chromosome segregation	0.02379324		
	nuclear division	0.02379324		

MODULE	TOP TERMS (Max 10)	Q VAL	GENES	TERMS
M4	regulation of cell-cell adhesion mediated by integrin	0.00300869	42	68
	cell-cell adhesion mediated by integrin	0.00407265		
	extracellular matrix organization	0.00529708		
	plasminogen activation	0.00823837		
	regulation of leukocyte activation	0.00899977		
	regulation of cell activation	0.01030412		
	extracellular matrix disassembly	0.01030412		
	regulation of cell adhesion mediated by integrin	0.01030412		
	extracellular structure organization	0.01052114		
	positive regulation of cell-cell adhesion	0.01240028		
M5	negative regulation of smooth muscle cell migration	0.00349773	27	65
	cellular response to retinoic acid	0.00432434		
	regulation of smooth muscle cell migration	0.00583972		
	negative regulation of smooth muscle cell proliferation	0.00605409		
	smooth muscle cell migration	0.00669382		
	positive regulation of actin filament bundle assembly	0.00690591		
	response to retinoic acid	0.00711742		
	muscle cell migration	0.00711742		
	regulation of actin filament bundle assembly	0.01211226		
	actin cytoskeleton reorganization	0.01241881		
M6	monovalent inorganic cation transport	0.00349773	2	1
M7	carbohydrate derivative catabolic process	0.00407265	5	1
M8	organophosphate biosynthetic process	0.00669382	20	13
	nucleotide biosynthetic process	0.01769321		
	nucleoside phosphate biosynthetic process	0.01776932		
	cellular amino acid metabolic process	0.02061688		
	ncRNA metabolic process	0.03876460		
	nucleotide metabolic process	0.03980843		
	nucleoside phosphate metabolic process	0.04036902		
	translation	0.04264931		
	peptide biosynthetic process	0.04445730		
	nucleobase-containing small molecule metabolic process	0.04826356		
M9	intracellular receptor signaling pathway	0.02615863	21	5
	positive regulation of protein kinase activity	0.05247830		
	tube morphogenesis	0.05671801		
	positive regulation of kinase activity	0.05712028		
	tube development	0.06273924		

MODULE	TOP TERMS (Max 10)	Q VAL	GENES	TERMS
M10	head development	0.04623470	78	9
	generation of neurons	0.05671801		
	central nervous system development	0.06087637		
	neurogenesis	0.06273924		
	cell morphogenesis involved in differentiation	0.07418971		
	anion transmembrane transport	0.08690943		
	regulation of neurogenesis	0.08759198		
	regulation of ion transmembrane transport	0.09533357		
	regulation of nervous system development	0.09598894		
