# Supplementary material for: Whole‐genome methylation analysis of aging human tissues identifies age‐related changes in developmental and neurological pathways
Source: Aging Cell. 2023 Jun 12;22(7):e13847. doi: 10.1111/acel.13847 (PMC10352543; doi:10.1111/acel.13847)
Supplement: Supplementary file 12 — Table S11. [file ACEL-22-e13847-s001.docx]

Table S1. Read depth and coverage for all WGBS samples.

Table S2. Linear regression results from all muscle samples.

Table S3. Linear regression results from all monocyte samples.

Table S4. Muscle differentially methylated regions.

Table S5. Monocyte differentially methylated regions.

Table S6. Annotations for muscle aDMRs.

Table S7. Annotations for monocyte aDMRS.

Table S8. HumanBase analysis results.

Table S9. Muscle and monocyte KEGG analysis results.

Table S10. Common aDMPs for all analysis methods for muscle and monocyte.
